# Decomposition of Zero-Dimensional Persistence Modules via Rooted Subsets

**DOI:** 10.1007/s00454-024-00700-7

**Published:** 2024-11-16

**Authors:** Ángel Javier Alonso, Michael Kerber

**Affiliations:** Institute of Geometry, Graz University of Technology, Graz, Austria

**Keywords:** Multiparameter persistence homology, Clustering, Decomposition of persistence modules, Elder rule, 55N31

## Abstract

We study the decomposition of zero-dimensional persistence modules, viewed as functors valued in the category of vector spaces factorizing through sets. Instead of working directly at the level of vector spaces, we take a step back and first study the decomposition problem at the level of sets. This approach allows us to define the combinatorial notion of *rooted subsets*. In the case of a filtered metric space *M*, rooted subsets relate the clustering behavior of the points of *M* with the decomposition of the associated persistence module. In particular, we can identify intervals in such a decomposition quickly. In addition, rooted subsets can be understood as a generalization of the elder rule, and are also related to the notion of constant conqueror of Cai, Kim, Mémoli and Wang. As an application, we give a lower bound on the number of intervals that we can expect in the decomposition of zero-dimensional persistence modules of a density-Rips filtration in Euclidean space: in the limit, and under very general circumstances, we can expect that at least 25% of the indecomposable summands are interval modules.

## Introduction

Multiparameter persistent homology is an active research area in topological data analysis. The motivation is that in many datasets there are multiple parameters that deserve attention in a multiscale analysis [11, 18, 31]. Concretely, when analyzing point clouds, we want to consider the distances between points, but also potentially remove points of low density.

A central object of persistent homology is the *persistence module*, which tracks algebraically how the topological features of the data change as we move through the parameter space. In the single-parameter case, every persistence module decomposes into a collection of intervals, called the *persistence barcode* [20], where each interval represents the lifetime of a topological feature in the data. In the multiparameter setting, there is a generalized notion of interval, which again represents the lifetime of a topological feature, but decomposing a multiparameter persistence module into intervals is not always possible, and one might be left with non-interval indecomposable persistence modules that lead to complications, both theoretically [12, 13, 18, 32] and computationally [1, 4, 23].

In fact, the classification of such indecomposable persistence modules is thought to be out of reach: certain involved posets are of *wild representation type*, even when accounting for certain simplifications [2]. Moreover, infinite families of complicated indecomposable persistence modules can be realized by simple geometric constructions [13], and, most recently, it has been shown in [3] that multiparameter persistence modules are, generically, close to being indecomposable, under the interleaving metric (we refer to [3] for a precise statement).

Still, the mentioned complications do not imply that the persistence modules that come up in practice are close to indecomposable, or that they are not decomposable into intervals. Indeed, is the decomposition of multiparameter persistence modules as badly behaved in practice as we can expect in theory? The authors of [2] and those of [3] state similar questions.

As an initial test, we computed the decomposition of persistence modules for a standard zero (homological) dimension construction, using a prototypical implementation of the algorithm by Dey and Xin [23] (this implementation will be discussed in another paper). As we see in Table 1, the assumption that persistence modules can be decomposed completely into intervals seems to be false most of the time, at least in this setting. However, Table 1 also shows that in all tested instances, *most* indecomposable summands are indeed intervals.

**Table 1:** Number of intervals in the decomposition of zero-dimensional persistence modules for density-Rips filtrations

Sample	Densities	100 points					500 points				
		Run 1	2	3	4	5	Run 1	2	3	4	5
xclustered	kde	100	98	95	98	98	474	487	478	479	479
uniform	kde	88	88	86	88	86	444	447	433	453	457
clustered	random	77	86	87	88	76	397	381	390	380	386
uniform	random	76	79	75	75	70	376	361	366	355	377

We tried both *clustered* samples where the points were sampled by a multivariate Gaussian distribution around 5 peaks, and *uniform* samples in the unit square. The density parameter was computed via a Gaussian kernel density estimate (*kde*) or a *random* density was assigned. The table shows the number of intervals for 5 independent test runs; for *n* points, the module is interval-decomposable if the number of intervals is *n*. This only happens for one run

This begs the question whether we can provably expect many intervals in general. In addition, knowledge of the intervals can greatly simplify and speed up computational tasks for persistence modules: for instance, a popular way to analyze 2-parameter persistence modules is by considering 1-dimensional restrictions, so-called slices, resulting in a parameterized family of persistent barcodes [29, 31, 33, 35]. Every interval of the 2-dimensional persistence module gives one bar in the barcode of the slice, by intersecting the slice with the interval. Thus, by knowing the intervals, existing algorithms can focus on the non-interval “core” of the problem, which is typically of much smaller size.

The practical problem of the described approach is that decomposing a multiparameter persistence module is costly, despite ongoing efforts [23]. However, to leverage the knowledge of intervals there is no need to compute a total decomposition, or to even identify all intervals. It suffices to have a method to “peel off” intervals from a persistence module quickly. Thus, we pose the question whether there exist methods that work very fast in practice and still are capable of detecting many intervals.

### Contributions

We focus on the case of zero-dimensional persistence modules. Already this case is of practical interest because of its connection to hierarchical clustering methods (see Sect. 1.2), and has received attention recently [2, 10, 14, 33]. In this context, we give some answers to the questions stated above:

For a point cloud *M*, a *nearest neighbor pair* is a pair $(x, y)\in M\times M$ such that *y* is the nearest neighbor of *x* and *x* is the nearest neighbor of *y* (breaking ties with a fixed total order). The theory we develop says that for a zero-dimensional persistence module of the density-Rips bifiltration (for any density estimation function), there are at least as many intervals as there are nearest neighbor pairs in *M*. These intervals are easily determined by the nearest neighbor pairs, and we refer to them as *NN-intervals*. Since all nearest neighbor pairs can be computed in $O(n\log n)$ time [19, 37], this yields a fast method to compute all NN-intervals of the decomposition. Moreover, we can expect many NN-intervals: using previous results on nearest neighbor graphs, we show that if *M* is sampled independently from an arbitrary, almost continuous density function, at least a quarter of the summands in the decomposition are intervals as $n\rightarrow \infty$. To our knowledge, this is the first result proving a non-constant lower bound on the number of intervals in a decomposition.

To arrive at this result, we use the following main idea: Instead of studying the decomposition of the persistence module directly in the category of (graded) vector spaces, we work in the category of *persistent sets*, whose objects can be interpreted as a two-parameter hierarchical clustering. The decomposition of a persistence module is governed by its idempotent endomorphisms, so we look for idempotent endomorphisms not of persistence modules, but of persistent sets, which are simpler. We show that such idempotent endomorphisms can be translated into *rooted subsets*, which are subsets of points that get consistently merged with a fixed point in the hierarchical clustering. Moreover, rooted subsets with a single element correspond to intervals in the associated persistence module.

Instead of peeling off intervals from the persistence module, we peel off rooted subsets from the persistent set. The advantage is that the remaining structure is still a hierarchical clustering, and the process can be iterated.

### Related Work

Multiparameter persistent sets and zero-dimensional persistence modules, as we will study them here, are related to a multiparametric approach to the clustering problem first considered by Carlsson and Mémoli [16]. The need for multiple parameters, density and scale, is justified by an axiomatic approach to clustering [15, 17, 28]. The application of techniques from multiparameter persistence homology, like persistence modules and interleavings, to this setting has attracted attention recently [2, 14, 31, 33, 35].

Cai et al. [14] define a useful summary for zero-dimensional persistence modules coming from density-Rips, called the *elder-rule-staircode*, inspired by the elder rule [24]. They also introduce the related concept of constant conqueror, and they ask whether a constant conqueror induces an interval in the decomposition of the associated persistence module. We answer this question in the negative with Example 4.3, and, in contrast, we show that a *rooted generator*, as introduced here, does induce an interval in the decomposition (Corollary 3.9).

Brodzki et al. [10] also study the decomposition of zero-dimensional persistence modules. They identify a class of persistence modules, called *semi-component modules*, that may appear as summands in the decomposition of zero-dimensional modules, but that are still hard to classify. Their methods have been of great inspiration, and in Theorem 5.1 we give another proof, within the theory we develop, of a theorem of theirs.

## Preliminaries

### Persistent Sets and Persistence Modules

In what follows, we let *P* be a finite partially ordered set (a finite *poset*), which we view as a category. A **persistence module** (over *P*) is a functor from *P* to the category $\textsf{Vec}$ of finite dimensional vector spaces, over a fixed field *K*. Such a functor $F:P\rightarrow \textsf{Vec}$ associates to each **grade** $p\in P$ a finite dimensional vector space $F_{p}$ and to each morphism $p\le q$ in *P* a linear map $F_{p\rightarrow q}:F_{p}\rightarrow F_{q}$, in such a way that $F_{p\rightarrow p} = \textrm{id}$ and composition is preserved. We see persistence modules as the objects of the functor category $\textsf{Vec}^{P}$, where natural transformations are the morphisms. In this sense, a morphism $f:F\rightarrow G$ of persistence modules is a family of maps $\{f_{p}:F_{p}\rightarrow G_{p}\}_{p\in P}$ such that for every two $p\le q$ the following diagram commutes 
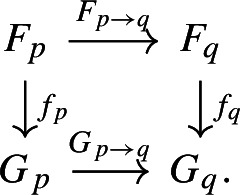


Similarly, a **persistent set** (over *P*) is a functor from *P* to $\textsf{Set}$, the category $\textsf{Set}$ of finite sets, and morphisms of persistent sets are natural transformations as above.

We can obtain a persistence module from a persistent set by the application of the **linearization functor** $\textsf{Set}\rightarrow \textsf{Vec}$ that takes each set to the free vector space generated by it. This linearization functor induces a functor ${\mathcal {L}}:\textsf{Set}^{P}\rightarrow \textsf{Vec}^{P}$ by postcomposition.

### From Geometry to Persistent Sets

Let (*M*, *d*) be a finite metric space, and consider a function $f:M\rightarrow {\mathbb {R}}$. We can understand *f* as an assignment of a *density* to each of the points of *M*; that is, a density estimation function [36]. We assume that *f* assigns lower values to points of *higher* density. Following [14], we call the triple (*M*, *d*, *f*) an **augmented metric space**. We construct a persistent set, the **density-Rips persistent set** of (*M*, *d*, *f*), that tracks how the clustering of points of *M* changes as we change the density and scale parameters, in a sense that we make precise shortly.

First, for a fixed scale parameter $\varepsilon \ge 0$, we define the **geometric graph of** *M* **at** $\varepsilon$, denoted by ${\mathcal {G}}_{\varepsilon }(M)$, as the undirected graph on the vertex set *M* and edges (*x*, *y*) where $d(x, y)\le \varepsilon$. The connected components of ${\mathcal {G}}_{\varepsilon }(M)$, as $\varepsilon$ goes from 0 to $\infty$, form the clusters of the dendrogram obtained via the single-linkage clustering method.

To introduce the density, for each $\sigma \in {\mathbb {R}}$ we let $M_{\sigma } {:}{=} \{x\in M| f(x) \le \sigma \} \subseteq M$ be the metric subspace of points with (co)density below $\sigma$. For any two $\sigma \le \sigma '$, $M_{\sigma }\subseteq M_{\sigma '}$ and by taking each $(\varepsilon , \sigma )$ to the graph ${\mathcal {G}}_{\varepsilon }(M_{\sigma })$, we obtain a functor ${\mathcal {G}}(M, f):{\mathbb {R}}_{\ge 0}\times {\mathbb {R}}\rightarrow \textsf{Graph}$, where the order in ${\mathbb {R}}_{\ge 0}\times {\mathbb {R}}$ is given by $(\varepsilon ,\sigma )\le (\varepsilon ',\sigma ')$ if and only if $\varepsilon \le \varepsilon '$ and $\sigma \le \sigma '$. We then consider the **connected components functor** $\pi _{0}:\textsf{Graph}\rightarrow \textsf{Set}$, that takes each graph to its set of connected components. In this way, we obtain a functor $\pi _{0}\circ {\mathcal {G}}(M, f):{\mathbb {R}}_{\ge 0}\times {\mathbb {R}}\rightarrow \textsf{Set}$.

#### Remark 2.1

The linearized persistence module ${\mathcal {L}}(\pi _{0}\circ {\mathcal {G}}(M, f)):{\mathbb {R}}_{\ge 0}\times {\mathbb {R}}\rightarrow \textsf{Vec}$ is isomorphic to the persistence module obtained by applying zero-dimensional homology at graph level, $H_{0}\circ {\mathcal {G}}(M, f):{\mathbb {R}}_{\ge 0}\times {\mathbb {R}}\rightarrow \textsf{Vec}$. In this sense, the construction we have described is the zero-dimensional level of the density-Rips filtration, which is standard in multiparameter persistent homology (see [5, 18] and also [14]).

We can understand the functor $\pi _{0}\circ {\mathcal {G}}(M, f):{\mathbb {R}}_{\ge 0}\times {\mathbb {R}}\rightarrow \textsf{Set}$ as a persistent set $S:P\rightarrow \textsf{Set}$ indexed by a finite grid $P\subseteq {\mathbb {R}}_{\ge 0}\times {\mathbb {R}}$ in the following way. We consider the set of distances $D {:}{=} \{d(x, y)| x, y\in M\}$ and densities $T{:}{=} \{f(x)| x\in M\}$, and define a finite grid $P{:}{=} D\times T\subset {\mathbb {R}}_{\ge 0}\times {\mathbb {R}}$. Finally, we define the persistent set $S:P\rightarrow \textsf{Set}$ by taking each $(\varepsilon ,\sigma )\in P$ to $(\pi _{0}\circ {\mathcal {G}}(M, f))_{(\varepsilon ,\sigma )}$, and similarly for the morphisms.

#### Definition 2.2

Let (*M*, *d*, *f*) be an augmented metric space. We define its **density-Rips persistent set** as the functor $S:P\rightarrow \textsf{Set}$, constructed as above.

### Decomposition of Persistence Modules

We can study persistence modules via their decomposition. For two persistence modules *F* and *G* their direct sum $F\oplus G$ is the persistence module given by taking direct sums pointwise, $(F\oplus G)_{p} = F_{p}\oplus G_{p}$. A persistence module is **indecomposable** if $F \cong F_{1}\oplus F_{2}$ implies that either $F_{1} = 0$ or $F_{2} = 0$. Since persistence modules are actual modules (see, for instance, [8, Lem. 2.1]), by the Krull–Schmidt theorem, a decomposition of a persistence module $F = F_{1}\oplus F_{2}\oplus \dots F_{n}$ into indecomposable summands is unique up to permutation and isomorphism of the summands.

A subposet $I\subset P$ is called an **interval** if it is non-empty, connected and convex, meaning that for any two $i,j\in I$ and any $l\in P$, if $i\le l \le j$ then $l\in I$. The **interval module supported on the interval** $I\subset P$, denoted ${\mathcal {I}}(I):P\rightarrow \textsf{Vec}$, is the indecomposable (by, e.g. [7, Prop. 2.2]) persistence module given by

$$\begin{aligned} {\mathcal {I}}(I)_{p} = {\left\{ \begin{array}{ll} K, &  \text {if}\, p\in I,\\ 0, &  \text {otherwise,} \end{array}\right. } \quad \text {with internal maps}\quad {\mathcal {I}}(I)_{p\rightarrow q} = {\left\{ \begin{array}{ll} \textrm{id}, &  \text {if}\, p,q\in I,\\ 0, &  \text {otherwise.} \end{array}\right. } \end{aligned}$$

If *P* is a totally ordered set, every persistence module over *P* decomposes as a direct sum of interval modules [6], but such a nice decomposition does not exist in general for other posets.

### Decomposition and Endomorphisms

A direct sum $X = X_{1}\oplus X_{2}$ of persistence modules is characterized up to isomorphism by morphisms $\iota _{i}:X_{i}\rightarrow X$ and $\pi _{i}:X\rightarrow X_{i}$ for $i = 1,2$ such that $\pi _{i}\circ \iota _{i} = \textrm{id}_{X_{i}}$ and $\iota _{1}\circ \pi _{1} + \iota _{2}\circ \pi _{2} = \textrm{id}_{X}$ (see, for instance, [30]). In this case, for each $i = 1, 2$, the maps $\iota _{i}$ and $\pi _{i}$ induce an endomorphism $\varphi _{i} {:}{=} \iota _{i}\circ \pi _{i}$ of *X*. Such an endomorphism $\varphi _{i}:X\xrightarrow {\pi _{i}} X_{i}\xrightarrow {\iota _{i}} X$ is also split:

#### Definition 2.3

In any category, we say that an endomorphism $\varphi :X\rightarrow X$ is **split** if there exists an object *Y* and a factorization $\varphi :X\xrightarrow {\pi } Y\xrightarrow {\iota } X$ such that $\pi \circ \iota = \textrm{id}_{Y}$.

We will use the following standard fact about split endomorphisms:

#### Lemma 2.4

Let $\varphi :X\rightarrow X$ be a split endomorphism that has two factorizations $X\xrightarrow {\pi } Y \xrightarrow {\iota } X$ and $X\xrightarrow {\pi '} Y'\xrightarrow {\iota '} X$ with $\pi \circ \iota = \textrm{id}_{Y}$ and $\pi '\circ \iota ' = \textrm{id}_{Y'}$. Then *Y* and $Y'$ are isomorphic.

#### Proof

We show that the compositions $\pi '\circ \iota :Y \xrightarrow {\iota } X \xrightarrow {\pi '} Y'$ and $\pi \circ \iota ':Y' \xrightarrow {\iota '} X \xrightarrow {\pi } Y$ are inverse to each other. Using the definition of a split endomorphism we compute

$$\begin{aligned} (\pi \circ \iota ') \circ (\pi ' \circ \iota ) = \pi \circ (\iota ' \circ \pi ') \circ \iota = \pi \circ \varphi \circ \iota = \pi \circ \iota \circ \pi \circ \iota = \textrm{id}_{Y}, \end{aligned}$$

and, similarly, $(\pi ' \circ \iota ) \circ (\pi \circ \iota ') = \textrm{id}_{Y'}$. We conclude that *Y* and $Y'$ are isomorphic. $\square$

Every split endomorphism $\varphi :X\xrightarrow {\pi } Y\xrightarrow {\iota } X$ is also **idempotent**, meaning that $\varphi \circ \varphi = \varphi$. Moreover, in our categories of interest, namely persistent sets $\textsf{Set}^{P}$ and persistence modules $\textsf{Vec}^{P}$, every idempotent endomorphism splits through its **image**, see below. In these two categories, we define the image of a morphism *f*, ${{\,\textrm{img}\,}}f$, by taking the image pointwise, that is, $({{\,\textrm{img}\,}}f)_{p} = f_{p}(S_{p})$. The following two lemmas are standard.

#### Lemma 2.5

Let $\varphi :X\rightarrow X$ be an idempotent endomorphism in $\textsf{Vec}^{P}$ or $\textsf{Set}^{P}$. Then *f* splits through its image: there exists a factorization $f:X\xrightarrow {\pi } {{\,\textrm{img}\,}}\varphi \xrightarrow {\iota } X$ with $\pi \circ \iota = \textrm{id}_{{{\,\textrm{img}\,}}\varphi }$.

#### Proof

In any abelian category, like $\textsf{Vec}^{P}$, every morphism $f:X\rightarrow Y$ has a factorization $X\xrightarrow {\pi } {{\,\textrm{img}\,}}f \xrightarrow {\iota } Y$ where $\pi$ is an epimorphism and $\iota$ a monomorphism (see [30, Prop. VIII.3.1]). It is easy to check that the same happens in $\textsf{Set}$ and $\textsf{Set}^{P}$.

Now, consider an idempotent endomorphism $\varphi :X\rightarrow X$ and its epi-mono factorization $\varphi = \iota \circ \pi$ as above. Since it is idempotent, we have $\varphi \circ \varphi = (\iota \circ \pi )\circ (\iota \circ \pi ) = \iota \circ \pi$. Since $\pi$ is an epimorphism and is right cancellable, and since $\iota$ is a monomorphism and is left cancellable, from $\iota \circ (\pi \circ \iota )\circ \pi = \iota \circ \pi$ we can obtain $\pi \circ \iota = \textrm{id}_{{{\,\textrm{img}\,}}\varphi }$, as desired. $\square$

#### Lemma 2.6

Let $F:P\rightarrow \textsf{Vec}$ be a persistence module, and let $\varphi :F\rightarrow F$ be an idempotent endomorphism. Then *F* decomposes as ${{\,\textrm{img}\,}}(\textrm{id}_{F} - \varphi )\oplus {{\,\textrm{img}\,}}\varphi$.

#### Proof

Applying Lemma 2.5 to $\varphi$, we have a factorization $\varphi :F\xrightarrow {\pi } {{\,\textrm{img}\,}}\varphi \xrightarrow {\iota } F$ with $\iota \circ \pi = \varphi$ and $\pi \circ \iota = \textrm{id}$. In turn, $\textrm{id}-\varphi$ is also an idempotent, which splits and satisfies $\textrm{id}- \varphi + \varphi = \textrm{id}$, which, by the characterization of the direct sum (the paragraph above Definition 2.3), yields a decomposition of the form ${{\,\textrm{img}\,}}(\textrm{id}- \varphi )\oplus {{\,\textrm{img}\,}}\varphi$. $\square$

## Endomorphisms of Persistent Sets and Rooted Subsets

As seen above, the decomposition of a persistence module is intimately related to its idempotent endomorphisms. Our main idea is that, when studying the decomposition of persistence modules of the form ${\mathcal {L}}S$, for a persistent set $S:P\rightarrow \textsf{Set}$, we look for idempotent endomorphisms of *S* and study their image under the linearization functor ${\mathcal {L}}$.

### Definition 3.1

Given a persistent set *S*, a **generator** is a pair $(p_{x}, x)$ with $x\in S_{p_{x}}$ such that *x* is not in the image of any morphism $S_{q\rightarrow p_{x}}$ for any $q < p_{x}$. When it is clear, we will often suppress the grade $p_{x}$ from the notation, and directly write that $x\in S_{p_{x}}$ is a generator.

There is an induced preorder on the generators of *S*: for two generators $x\in S_{p_{x}}$ and $y\in S_{p_{y}}$ we say that $(p_{x}, x) \le (p_{y}, y)$ if and only if $p_{x}\le p_{y}$. This relation might not be antisymmetric, and so in general the preordered set of generators is not a poset.

Generators are useful because an endomorphism $\varphi$ of a persistent set $S:P\rightarrow \textsf{Set}$ is uniquely determined by the image of its generators: for each $z\in S_{q}$ we have $\varphi _{q}(z) = S_{p_{x}\rightarrow q}\circ \varphi _{p_{x}}(x)$ for some generator $x\in S_{p_{x}}$, by the commutativity property.

In linear algebra, an idempotent endomorphism can be thought as a projection onto its image, that is, onto its fixed points. This point of view and the concept of generators above motivates the following definition, which plays a fundamental role in our work, and where the fixed points are the “roots”.

### Definition 3.2

A **rooted subset** *A* is a non-empty subset of the generators of *S* such that there exists an idempotent endomorphism $\varphi$ of *S* whose set of generators that are not fixed is precisely *A*. If a rooted subset is a singleton, $A = \{x\}$, we say that *x* is a **rooted generator**.

**Fig. 1:**
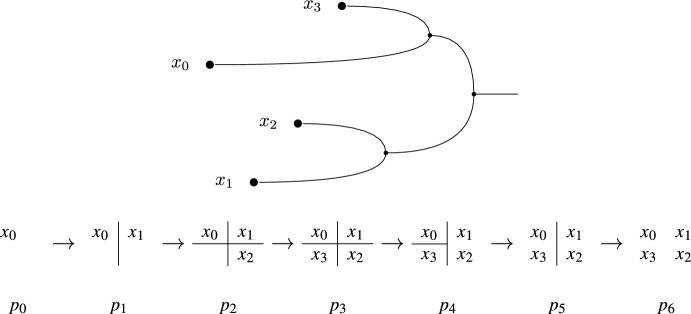
A persistent set $S:P\rightarrow \textsf{Set}$ over a totally ordered set *P* of 7 elements: $p_{0}< p_{2}< \cdots < p_{6}$. Each set $S_{p_{i}}$ is a partition of $\{x_{0}, x_{1}, x_{2}, x_{3}\}$, which is represented in the bottom part of the figure: $x_{i}$ and $x_{j}$ are in the same block if they are not separated by a line. Each arrow $S_{p_{i}\rightarrow p_{j}}$ takes each element of $S_{p_{i}}$ to the element of $S_{p_{j}}$ that contains it. In the upper part, *S* is represented as a *merge tree*

### Example 3.3

Let us consider the persistent set $S:P\rightarrow \textsf{Set}$ of Fig. 1. In this case the poset *P* is totally ordered. There are four generators, $(p_{i}, \{x_{i}\})$ for each $i=0,\dots , 3$. Here, $(p_{3}, \{x_{3}\})$ is a rooted generator: we can define an idempotent endomorphism $\varphi :S\rightarrow S$ with $\varphi _{p_{3}}(\{x_{3}\}) = S_{p_{0}\rightarrow p_{3}}(\{x_{0}\})$, and the identity on the other generators. But $(p_{1}, \{x_{1}\})$ is not a rooted generator: any idempotent endomorphism of *S* that does not fix $x_{1}$ also does not fix $x_{2}$.

### Remark 3.4

In the case of an augmented metric space (*M*, *d*, *f*) and its density-Rips persistent set *S* of Definition 2.2 there exists a bijection between the points of *M* and the generators of *S*. A point $x\in M$ first appears in the graph ${\mathcal {G}}_{0}(M_{f(x)})$, where *x* is always its own connected component. In what follows, we will often identify a point $x\in M$ with its generator $x\in S_{p_{x}}$. In this sense, we can understand an endomorphism of *S* as an endomorphism of the set of points that is compatible with the connected components of all graphs ${\mathcal {G}}_{\varepsilon }(M_{\sigma })$.

We are especially interested in persistent sets obtained from (augmented) metric spaces, and our objective is to relate rooted generators to the geometry of these objects. Considering an augmented metric space (*M*, *d*, *f*) and its density-Rips persistent set, Proposition 3.5 characterizes rooted generators by the clustering behavior of the points of *M*.

### Proposition 3.5

Let (*M*, *d*, *f*) be an augmented metric space and consider a point $x\in M$. If there exist some other point $y\in M$ such that

(i) $f(y)\le f(x)$ (i.e. *y* is “denser” than *x*), and
(ii) whenever *x* is in a cluster of more than one point, $y\in M$ is in the same cluster: that is, for every ${\mathcal {G}}_{\varepsilon }(M_{\sigma })$, if *x* is path-connected to some other point then *x* is path-connected to *y*,

then the generator $(p_{x}, x)$ of the density-Rips persistent set *S* of *M* is a rooted generator.

Conversely, if *x* is a rooted generator of *S*, then there exists a point $y\in M$ that satisfies conditions (i) and (i) above.

### Proof

Before going into the proof, recall that, by the way we construct *S* and the inclusion $P\hookrightarrow {\mathbb {R}}_{\ge 0}\times {\mathbb {R}}$, for each $q\in P$ there is an associated graph ${\mathcal {G}}_{\varepsilon }(M_{\sigma })$, for some $(\varepsilon , \sigma )\in {\mathbb {R}}_{\ge 0}\times {\mathbb {R}}$. Each element $z\in S_{q}$ is a connected component of this graph ${\mathcal {G}}_{\varepsilon }(M_{\sigma })$, and the generators $x\in S_{p_{x}}$ such that $S_{p_{x}\rightarrow q}(x) = z$ are precisely the points in that connected component.

The first part follows from Proposition 3.6, which proves it in more generality.

For the converse, let $\varphi$ be an idempotent of *S* whose only generator that is not fixed is $x\in S_{p_{x}}$. This means that there exists a generator $y\in S_{p_{y}}$, different from *x*, such that $\varphi _{p_{x}}(x) = S_{p_{y}\rightarrow p_{x}}(y)$. And clearly $\varphi _{p_{z}}(z) = z$ for any other generator $z\in S_{p_{z}}$. From the fact that $\varphi _{p_{x}}(x) = S_{p_{y}\rightarrow p_{x}}(y)$ we deduce that $f(y)\le f(x)$, since $p_{y}\le p_{x}$ in *P*. To see that the second condition holds, pick a $q\ge p_{x}$ and suppose that there exists a generator $w\in S_{p_{w}}$ such that $S_{p_{x}\rightarrow q}(x) = S_{p_{w}\rightarrow q}(w)$. This means that in the graph ${\mathcal {G}}_{\varepsilon }(M_{\sigma })$ associated to *q* both *x* and *w* are in the same connected component, and we claim that *y* is also in this component. Indeed, by the definition of $\varphi$ we have $S_{p_{x}\rightarrow q}\circ \varphi _{p_{x}}(x) = S_{p_{y}\rightarrow q}(y) = S_{p_{w}\rightarrow q}(w)$. $\square$

### Proposition 3.6

Let (*M*, *d*, *f*) be an augmented metric space. If for a set of points $A\subset M$ there exists a point $y\not \in A$ such that for every $x\in A$, $f(y)\le f(x)$ and for each ${\mathcal {G}}_{\varepsilon }(M_{\sigma })$ either:

- *x* is path-connected to *y*, or
- the set of points that are path-connected to *x* is contained in *A*,

then the set of generators $\{(p_{x}, x) | x\in A\}$ is a rooted subset in the density-Rips persistent set *S* of *M*.

### Proof

To show that *A* is a rooted subset, we need to define an appropriate idempotent $\varphi$ of *S*. Recalling that an endomorphism is uniquely determined by the image of its generators, we define $\varphi$ by setting

1 $$\begin{aligned} \varphi _{p_{x}}(x) = {\left\{ \begin{array}{ll} S_{p_{y}\rightarrow p_{x}}(y), &  \hbox { if}\ x\in A,\\ x, &  \text {otherwise}, \end{array}\right. } \end{aligned}$$

for every generator $x\in S_{p_{x}}$. We need to show that $\varphi$ is indeed well-defined, which means that the image of $z\in S_{q}$, $\varphi _{q}(z) = S_{p_{x}\rightarrow q}\circ \varphi _{p_{x}}(x)$, is the same no matter the generator $x\in S_{p_{x}}$ we choose. Fix a $q\in P$ and a $z\in S_{q}$, and consider the set *G* of generators whose image in $S_{q}$ is *z*, $G{:}{=} \{(p_{x}, x) | p_{x}\le q,\ S_{p_{x}\rightarrow q}(x) = z\}$.

Then, to check that $\varphi$ is well-defined, for every two $(p_{x}, x), (p_{w}, w)\in G$ it must hold that

2 $$\begin{aligned} S_{p_{x}\rightarrow q}\circ \varphi _{p_{x}}(x) = S_{p_{w}\rightarrow q}\circ \varphi _{p_{w}}(w). \end{aligned}$$

If both $(p_{x}, x)$ and $(p_{w}, w)$ are not in *A*, or if both $(p_{x}, x)$ and $(p_{w}, w)$ are in *A*, then (2) above trivially holds, by the way we have defined $\varphi$ in (1).

Thus, the only interesting case is that only one of $(p_{x}, x)$ or $(p_{w}, w)$ is in *A*. Say that $(p_{x}, x)\in A$ and $(p_{w}, w)\not \in A$. Then, by assumption both *x* and *w* need to be path-connected to *y* at the graph ${\mathcal {G}}_{\varepsilon }(M_{\sigma })$ associated to *q*, which means that, as desired,

$$\begin{aligned} S_{p_{x}\rightarrow q}\circ \varphi _{p_{x}}(x) = S_{p_{y}\rightarrow q}(y) = S_{p_{w}\rightarrow q}(w) = S_{p_{w}\rightarrow q}\circ \varphi _{p_{w}}(w). \end{aligned}$$

Now, $\varphi$ is idempotent, because for every $x\in A$ we have $\varphi _{p_{x}}^{2}(x) = \varphi _{p_{x}}(S_{p_{y}\rightarrow p_{x}}(y)) = S_{p_{y}\rightarrow p_{x}}(\varphi _{p_{y}}(y)) = S_{p_{y}\rightarrow p_{x}}(y)$. And it is clear that the only generators that are not fixed by $\varphi$ are those in *A*. We conclude that, effectively, *A* is a rooted subset. $\square$

**Fig. 2:**
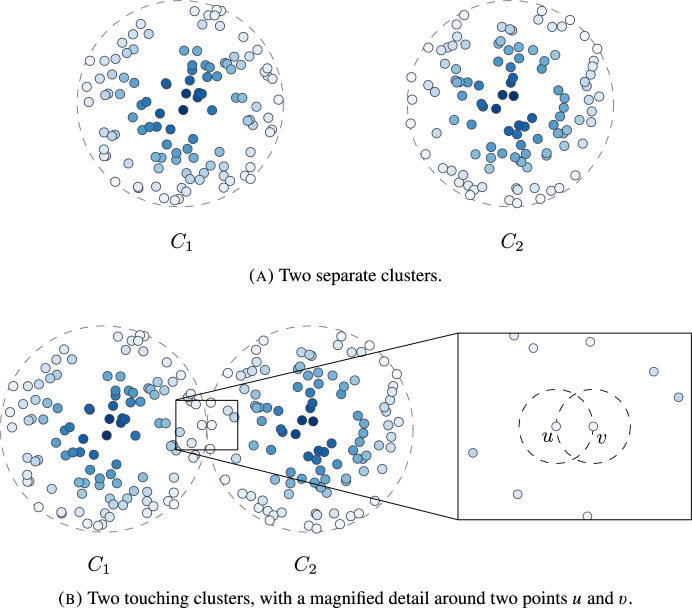
Point clouds of two clusters sampled from a multivariate Gaussian distribution, referenced in Example 3.7. Each point cloud *X* is turned into an augmented metric space by the function $g(x) = 1 - f(x)$, where *f* is the underlying probability density function. Points of darker color have lower values of *g*, that is, are *denser*

### Example 3.7

Consider the two clusters, $C_{1}$ and $C_{2}$, of the point cloud *X* of Fig. 2a. Let $x_{1}$ and $x_{2}$ be the densest points of $C_{1}$ and $C_{2}$, respectively. Since the clusters are separated by enough distance, at every graph ${\mathcal {G}}_{\varepsilon }(M_{\sigma })$, every point of $C_{1}$ is path-connected to $x_{1}$ before being path-connected to any point of $C_{2}$, and analogously for $C_{2}$ and $x_{2}$. Therefore, $C_{1} {\setminus } \{x_{1}\}$ and $C_{2} {\setminus } \{x_{2}\}$ are rooted subsets.

In Fig. 2b, the two clusters “touch”. The sets $C_{1}{\setminus }\{x_{1}\}$ and $C_{2}{\setminus }\{x_{2}\}$ are not rooted subsets. Note that $u\in C_{1}$ and $v\in C_{2}$, as in the magnified detail of the figure, are always path-connected before being path-connected to any other point. In fact, *u* is the nearest neighbor of *v* and vice versa, which is the behavior we look for in Sect. 5.

### Decomposition Induced by Rooted Subsets

As we have seen, rooted subsets are related to the clustering behavior of the points. They are also related to the decomposition of the linearized persistence module: they induce summands.

#### Theorem 3.8

Let $\varphi$ be an idempotent endomorphism of a persistent set *S*. Then the persistence module ${\mathcal {L}}S$ decomposes into

$$\begin{aligned} {{\,\textrm{img}\,}}(\textrm{id}_{{\mathcal {L}}S} - {\mathcal {L}}\varphi ) \oplus {\mathcal {L}}({{\,\textrm{img}\,}}\varphi ). \end{aligned}$$

#### Proof

Since $\varphi$ is idempotent, ${\mathcal {L}}\varphi$ is idempotent. By Lemma 2.6, this induces a decomposition

$$\begin{aligned} {\mathcal {L}}S\cong {{\,\textrm{img}\,}}(\textrm{id}- {\mathcal {L}}\varphi )\oplus {{\,\textrm{img}\,}}{\mathcal {L}}\varphi . \end{aligned}$$

It is left to show that ${{\,\textrm{img}\,}}{\mathcal {L}}\varphi \cong {\mathcal {L}}({{\,\textrm{img}\,}}\varphi )$. Applying Lemma 2.5 to ${\mathcal {L}}\varphi$, we have a factorization ${\mathcal {L}}\varphi :{\mathcal {L}}S\xrightarrow {\pi } {{\,\textrm{img}\,}}{\mathcal {L}}\varphi \xrightarrow {\iota } {\mathcal {L}}S$ with $\pi \circ \iota = \textrm{id}$. Applying Lemma 2.5 again, this time to $\varphi$, we have a factorization $\varphi :S\xrightarrow {\pi '} {{\,\textrm{img}\,}}\varphi \xrightarrow {\iota '} S$ with $\pi ' \circ \iota ' = \textrm{id}$. Now, split endomorphisms are preserved by every functor: in the diagram ${\mathcal {L}}S \xrightarrow {{\mathcal {L}}\pi '} {\mathcal {L}}({{\,\textrm{img}\,}}\varphi ) \xrightarrow {{\mathcal {L}}\iota '} {\mathcal {L}}S$ it holds ${\mathcal {L}}\iota '\circ {\mathcal {L}}\pi ' = {\mathcal {L}}\varphi$ and ${\mathcal {L}}\pi '\circ {\mathcal {L}}\iota ' = \textrm{id}$. Thus, the endomorphism ${\mathcal {L}}\varphi$ splits in two ways: 
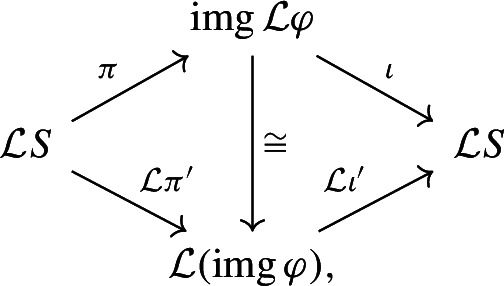
 where the middle arrow exists and is an isomorphism by Lemma 2.4, finishing the proof. $\square$

Combining the above theorem with the Krull–Schmidt theorem, we obtain the following:

#### Corollary 3.9

A rooted subset of a persistent set *S* induces a summand in the decomposition of ${\mathcal {L}}S$. A rooted generator $x\in S_{p_{x}}$ induces an interval summand, and all other summands can be obtained by decomposing ${\mathcal {L}}({{\,\textrm{img}\,}}\varphi )$, where $\varphi$ is the endomorphism associated to *x*.

This allows to iteratively *peel off* intervals of a persistence module of the form ${\mathcal {L}}S$: find a rooted generator of *S*, with associated idempotent $\varphi$, and continue considering ${{\,\textrm{img}\,}}\varphi$ instead of *S*. In the setting of an augmented metric space (*M*, *d*, *f*) and its density-Rips persistent set, the intervals that are peeled off are easily interpretable through the clustering behavior of the points *M*, by Proposition 3.5. Moreover, the conditions we describe actually happen in practice, as we see in Sect. 5.

### Neighborly Rooted Points

In fact, certain points of an augmented metric space (*M*, *d*, *f*) can be seen to be rooted by looking at the nearest neighbors, which will be useful in Sect. 5. In what follows we fix a total order on *M* compatible with the order induced by *f*. Recall that the **nearest neighbor** of *x* is the element $x'\ne x$ of minimum distance to *x*, where ties have been broken by the fixed total order on *M*.

#### Definition 3.10

Let (*M*, *d*, *f*) be an augmented metric space. An element *x* is **neighborly rooted** if its nearest neighbor $y\in M$ satisfies $f(y)\le f(x)$.

#### Lemma 3.11

With the notation as above, if a point $x\in M$ is neighborly rooted then *x* is a rooted generator in the density-Rips persistent set of (*M*, *d*, *f*).

#### Proof

It is clear that the nearest neighbor of *x* satisfies the conditions of Proposition 3.5. $\square$

#### Remark 3.12

We can identify all neighborly rooted points in the time it takes to solve the all-nearest-neighbor problem. Naturally, the all-nearest-neighbor problem can be solved in $O(n^{2})$, where *n* is the number of points, by checking all possible pairs. When the points are in Euclidean space, the running time can be improved to $O(n\log n)$ time [19, 37].

### Two Notable Intervals in the Decomposition

The concept of rooted generators allows us to prove that, in certain cases, we can find at least two intervals in the decomposition of ${\mathcal {L}}S$, as in Theorem 3.14. We first prove Theorem 3.13, which has already appeared in [10, Thm. 5.3], where the proof method is to directly construct an endomorphism of the persistence module, as we also do after composing with the linearization functor.

#### Theorem 3.13

Let *S* be a persistent set. Suppose that the preordered set of generators of *S* has a bottom $\bot$ (that is, one has $\bot \le x$ for any other generator *x*). Then the decomposition of ${\mathcal {L}}S$ consists of at least one interval, induced by $\bot$.

#### Proof

Let $\bot \in S_{p_{\bot }}$ be a bottom and let $x\in S_{p_{x}}$ be a generator of *S*. Since $\bot \in S_{p_{\bot }}$ is a bottom, we have $p_{\bot }\le p_{x}$. We can define an idempotent $\varphi :S\rightarrow S$ by $\varphi _{p_{x}}(x) = S_{p_{\bot }\rightarrow p_{x}}(\bot )$ for every generator $x\in S_{p_{x}}$ of *S*. This endomorphism is well-defined and its image has only one generator, namely $\bot$, and thus ${\mathcal {L}}({{\,\textrm{img}\,}}\varphi )$ is isomorphic to an interval module. $\square$

#### Theorem 3.14

Let (*M*, *d*, *f*) be an augmented metric space, and let $S:P\rightarrow \textsf{Set}$ be its density-Rips persistent set, as in Definition 2.2. If $|{M}| \ge 2$ then the decomposition of ${\mathcal {L}}S$ into indecomposable summands consists of at least two intervals.

#### Proof

Consider a point $\top \in M$ of maximal function value, that is, $f(\top )\ge f(x)$ for any other $x\in M$. Let *y* be the nearest neighbor of $\top$. Since $M_{f(\top )} = M_{\sigma }$ for any $\sigma \ge f(\top )$, it is clear that $\top$ and its nearest neighbor *y* satisfy the conditions of Proposition 3.5, and thus $\top$ is a rooted generator, yielding the first interval. For the second interval, we note that there is at least one point $\bot \in M$ of minimal density value and apply Theorem 3.13. $\square$

#### Example 3.15

**Fig. 3:**
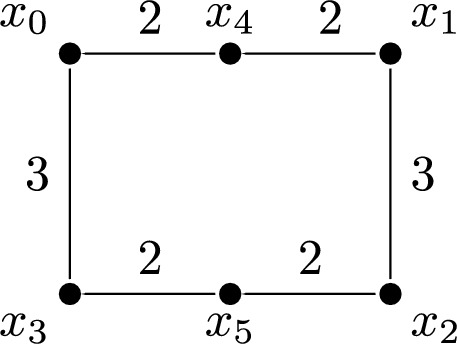
The augmented metric space (*M*, *d*, *f*) of Example 3.15, with $f(x_{i}) = i$. These are six points $\{x_{0}, \dots , x_{5}\}$ in the plane, where the distances are given by the numbers next to each line

**Fig. 4:**
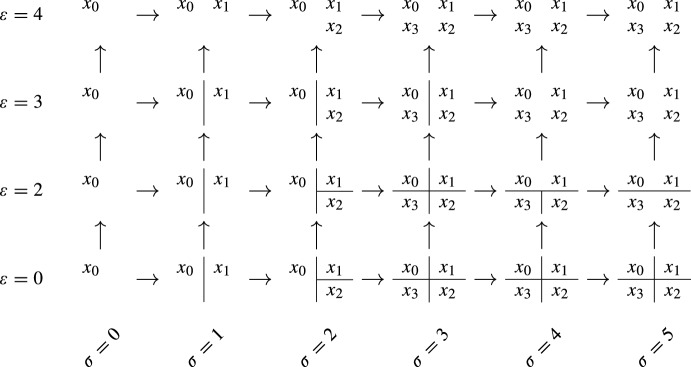
The persistent set $S:P\rightarrow \textsf{Set}$ of Example 3.15 obtained by taking the density-Rips persistence set of Fig. 3 and removing $x_{4}$ and $x_{5}$. Each node in the grid represents a partition of the $x_{i}$, where $x_{i}$ and $x_{j}$ are in the same partition if they are not separated by a line. The arrows are the functions that send the partition of $x_{i}$ in one node to the partition of $x_{i}$ in the other

Not every summand of an indecomposable decomposition can be obtained by taking rooted subsets and applying Corollary 3.9. As an example, consider the augmented metric space given by six points $\{x_{0}, \dots , x_{5}\}$ in the plane as in Fig. 3. Note that $x_{4}$ and $x_{5}$ are rooted in the associated density-Rips persistent set, and that they can be peeled off. After peeling, we obtain a persistent set $S:P\rightarrow \textsf{Set}$ with $P {:}{=} \{0, 2, 3, 4\}\times \{0, 1, 2, 3, 4, 5\}\subset {\mathbb {R}}^{2}$, which we describe in Fig. 4. This example is an adaptation of [14, Exam. 4.12], which is introduced in the context of *conquerors* that we discuss in Sect. 4.

We claim that the persistence module ${\mathcal {L}}S:P\rightarrow \textsf{Vec}$ decomposes into four summands, all of them interval modules. We denote these summands by $I_{0}, I_{1}, I_{2}$ and $I_{3}$, where each $I_{i}$ is associated to the generator $(p_{i}, x_{i})$ of *S*, where $p_{i} = (0, i)\in P$. For each $i = 0,\dots , 3$, we set $(I_{i})_{p} = 0$ for any $p < p_{i}$ and $(I_{i})_{p_{i}} = K$, and we define $\iota _{i}:I_{i}\rightarrow {\mathcal {L}}S$ by

$$\begin{aligned} (\iota _{0})_{p_{0}} (1)&= [x_{0}],&(\iota _{1})_{p_{1}} (1)&= [x_{1}] - [x_{0}],\\ (\iota _{2})_{p_{2}} (1)&= [x_{2}] - [x_{1}],&(\iota _{3})_{p_{3}} (1)&= [x_{3}] - [x_{0}] + [x_{1}] - [x_{2}]. \end{aligned}$$

The support of each $I_{i}$ are the grades $p\ge p_{i}$ such that $(({\mathcal {L}}S)_{p_{i}\rightarrow p}\circ (\iota _{i})_{p_{i}})(1)$ is not zero. It can be seen that these maps induce a decomposition ${\mathcal {L}}S \cong I_{0}\oplus I_{1}\oplus I_{2}\oplus I_{3}$.

However, no subset of the generators other than $\{x_{1}, x_{2}, x_{3}\}$ is rooted because each of the connected components given by $\{x_{1}, x_{0}\}$, $\{x_{1}, x_{2}\}$, $\{x_{2}, x_{3}\}$, and $\{x_{0}, x_{3}\}$ appear in *S*.

## Rooted Generators as a Generalization of the Elder Rule

### Single-Parameter Case

We now suppose that the finite poset *P* is totally ordered. In this setting, the theory of rooted generators allows us to recover the *elder rule* [24] (see also [14, 22]).

#### Proposition 4.1

Let *P* be a finite totally ordered poset and let $S:P\rightarrow \textsf{Set}$ be a persistent set. Suppose that *S* has at least two generators and that $S_{\top }$ is a singleton, where $\top$ is the maximum element of *P*. Then every maximal generator (in the preorder of Definition 3.1) is rooted.

#### Proof

Let $x\in S_{p_{x}}$ be a maximal generator, and define

$$\begin{aligned} I_{x} {:}{=} \{q\in P | q\ge p_{x} ~\hbox {and for any other generator}~ w\in S_{p_{w}}, S_{p_{w}\rightarrow q}(w) \ne S_{p_{x}\rightarrow q}(x)\}. \end{aligned}$$

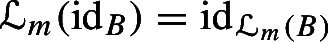


Since $p_{x}\in I_{x}$, $I_{x}$ is not empty, and we can consider the set $U\subset P$ of upper bounds of $I_{x}$. Moreover, since $S_{\top } = \{*\}$ and there are at least two generators by assumption, the set $U\setminus I_{x}$ is not empty. Let $\alpha$ be the least element in $U\setminus I_{x}$. By construction of $I_{x}$ and $U\setminus I_{x}$, there is a generator $y\in S_{p_{y}}$ such that $S_{p_{y}\rightarrow \alpha }(y) = S_{p_{x}\rightarrow \alpha }(x)$. Now, since *x* is maximal, it holds that $p_{y}\le p_{x}$, and we can define an idempotent $\varphi :S\rightarrow S$ by $\varphi _{p_{x}}(x) = S_{p_{y}\rightarrow p_{x}}(y)$, and $\varphi _{p_{z}}(z) = z$ for any other generator $z\in S_{p_{z}}$. Such an idempotent is well-defined by the way we have defined $\alpha$: if there is any other generator $w\in S_{p_{w}}$ such that $S_{p_{w}\rightarrow q}(w) = S_{p_{x}\rightarrow q}(x)$ then $\alpha \le q$ and also $S_{p_{y}\rightarrow q}(y) = S_{p_{x}\rightarrow q}(x)$. We conclude that *x* is rooted, as desired. $\square$

Thus, when *P* is a total order, we can decompose any persistence module ${\mathcal {L}}S$ by peeling off rooted generators, following Theorem 3.8 and by iteratively considering maximal generators. In this case, we can understand the decomposition of ${\mathcal {L}}S$ through the structure of *S*.

### Relation to Constant Conquerors

Let (*M*, *d*, *f*) be an augmented metric space. Cai et al. [14] define the concept of a constant conqueror as follows. First, define an ultrametric on *M*:

$$\begin{aligned} u(x, x'){:}{=} \min \{\varepsilon \in [0, \infty ) | x ~\hbox {and}~ x' \, \hbox {are path-connected in}\, {\mathcal {G}}_{\varepsilon }(M)\}. \end{aligned}$$

Now fix a total order $\prec$ on *M* and let $x\in M$ be a non-minimal element with respect to this order. A **conqueror** of *x* in *M* is another point $x'\in M$ such that (1) $x' \prec x$, and (2) for any $x''$ with $x'' \prec x$ one has $u(x, x')\le u(x, x'')$. Given a function $f:M\rightarrow {\mathbb {R}}$, a **conqueror function** of a non-minimal $x\in M$, with respect to $\prec$, is a function $c_{x}:[f(x), \infty )\rightarrow M$ that sends each $\sigma$ to a conqueror of *x* in $M_{\sigma }$. For the minimal element $\bot$ of *M* we define $c_{\bot }:[f(\bot ), \infty )\rightarrow M$ to be the constant function at $\bot$.

Also, in the same paper [14], given a point $x\in M$, and assuming that $f:M\rightarrow {\mathbb {R}}$ is injective, the authors define the **staircode** of *x* as the set given by

$$\begin{aligned} I_{x} {:}{=} \{(\varepsilon , \sigma )\in {\mathbb {R}}_{\ge 0}\times {\mathbb {R}}| x\in M_{\sigma } ~\hbox {and}~ x ~\hbox {is the oldest in}~ [x]_{(\varepsilon ,\sigma )}\}, \end{aligned}$$

where $[x]_{(\varepsilon , \sigma )}$ is the set of points that are path-connected to *x* in ${\mathcal {G}}_{\varepsilon }(M_{\sigma })$ and being the “oldest” means $f(x) < f(x')$ for any other $x'\in [x]_{(\varepsilon , \sigma )}$. The authors also define an analogous notion when *f* is not injective, which we do not reproduce here.

Finally, the authors ask the following question:

#### Question 4.2

Let (*M*, *d*, *f*) be an augmented metric spaceİf $x\in M$ has a constant conqueror function, is the interval module supported by $I_{x}$ a summand of its density-Rips persistence module?

If we replace constant conqueror by rooted generator then the answer is yes, by Corollary 3.9. The next example shows that the same cannot hold as originally stated in the question above.

#### Example 4.3

**Fig. 5:**
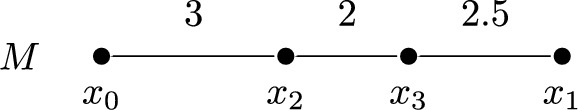
The augmented metric space (*M*, *d*, *f*) of Example 4.3, with $M\subset {\mathbb {R}}$ and $f(x_{i}) = i$

Consider the subset *M* of ${\mathbb {R}}$ given by the points $x_{0} = 0$, $x_{1} = 7.5$, $x_{2} = 3$ and $x_{3} = 5$. Under the metric induced by the Euclidean distance on ${\mathbb {R}}$, *M* is a metric space, and can be made into an augmented metric space by defining $f(x_{i}) = i$, see Fig. 5.

**Fig. 6:**
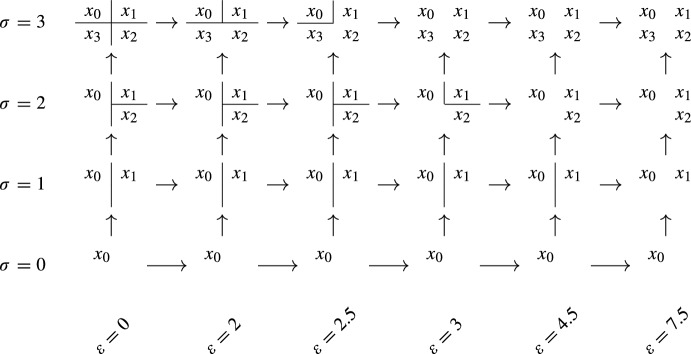
The density-Rips persistent set of Fig. 5

Consider the only total order $\prec$ on *M* compatible with *f*, $x_{0} \prec x_{1} \prec x_{2} \prec x_{3}$. The point $x_{1}$ has a constant conqueror: $x_{0}$ is the only candidate, and it is clear that, for every $i=1,\dots , 3$ and $x' \prec x_{1}$, $u_{i}(x_{1}, x_{0}) \le u_{i}(x_{1}, x')$, where $u_{i}$ is the ultrametric of $M_{i}$, precisely because $x_{0}$ is the only point that satisfies $x'\prec x_{1}$.

Let $S:P\rightarrow \textsf{Set}$ be the density-Rips persistent set constructed from the augmented metric space (*M*, *d*, *f*). Here, *P* is the subposet of ${\mathbb {R}}^{2}$ given by $\{0, 2, 2.5, 3, 4.5, 7.5\}\times \{0, 1, 2, 3\}$, where the first coordinate represents the distances and the second coordinate the densities. We picture *S* in Fig. 6. Now we proceed to decompose ${\mathcal {L}}S$. First, note that $x_{3}$ is a rooted generator, and consider an associated idempotent $\varphi :S\rightarrow S$. By Theorem 3.8, there is an interval $I{:}{=} {{\,\textrm{img}\,}}(\textrm{id}_{{\mathcal {L}}S} - {\mathcal {L}}\varphi )$ in the decomposition, and we can continue considering the persistent set ${{\,\textrm{img}\,}}\varphi$. In ${{\,\textrm{img}\,}}\varphi$, $x_{0}$ is a minimal generator. By Theorem 3.13 (and its proof) there is an idempotent $\psi :{{\,\textrm{img}\,}}\varphi \rightarrow {{\,\textrm{img}\,}}\varphi$ such that $I'{:}{=}{{\,\textrm{img}\,}}{\mathcal {L}}\psi$ is an interval. Applying Theorem 3.8 again, we obtain a decomposition of ${\mathcal {L}}S$ of the form

$$\begin{aligned} I\oplus I' \oplus {{\,\textrm{img}\,}}(\textrm{id}_{{\mathcal {L}}{{\,\textrm{img}\,}}\varphi } - {\mathcal {L}}\psi ). \end{aligned}$$

**Fig. 7:**
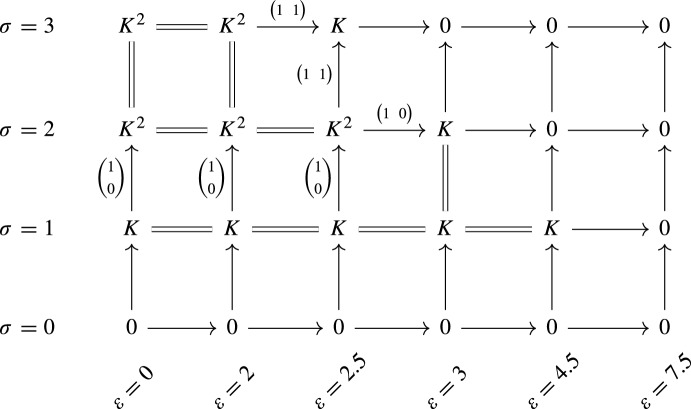
An indecomposable persistence module $F:P\rightarrow \textsf{Vec}$, as referenced in Example 4.3

By direct computation, it can be seen that ${{\,\textrm{img}\,}}(\textrm{id}_{{\mathcal {L}}{{\,\textrm{img}\,}}\varphi } - {\mathcal {L}}\psi )$ is isomorphic to the persistence module described in Fig. 7. Moreover, this persistence module is indecomposable, which can be checked by looking at its endomorphisms: a persistence module *F* is indecomposable if and only if every endomorphism of *F* is either nilpotent or an isomorphism (see [9, 10]).

Note that $x_{1}$ is not a rooted generator in ${\mathcal {L}}S$. In $M_{1}$, $x_{1}$ is its own connected component during $\varepsilon \in [0, 7.5)$, until $x_{0}$ joins the connected component. And in $M_{3}$ it is by itself during $\varepsilon \in [0, 2.5)$ and then joins the connected component of $x_{3}$, which is not connected to $x_{0}$ at that point. Similarly, $x_{2}$ is not rooted.

#### Remark 4.4

Note that in Condition (2) of the definition of conqueror, we require that $x'' \prec x$. This requirement measures part of the difference between constant conqueror function and rooted generator for augmented metric spaces. If we drop this requirement, denoting the resulting concept by ${\textbf {conqueror}}^{*}$, we suppose that *f* is injective, and that $\prec$ is compatible with the order induced by *f*, then a non-minimal, with respect to $\prec$, point $x\in M$ has a constant ${\textbf {conqueror}}^{*}$ function if and only if *x* is a rooted generator, as in Proposition 3.5.

## A Lower Bound on the Number of Expected Intervals

We apply the theory we have developed to the study of how a typical decomposition of a persistence module coming from density-Rips might look like. In particular, suppose we sample independently *n* points from a common density function *g*(*x*) in ${\mathbb {R}}^{d}$, obtaining a finite metric space $M\subset {\mathbb {R}}^{d}$. We can then consider the augmented metric space $(M, d_{M}, g)$, where *g*, rather than being an estimated density, is the true underlying density function. This setting resembles actual practice, but is more suitable to theoretical study. Let *S* be the density-Rips persistent set of *M*. Then, how many intervals can we expect in the decomposition of ${\mathcal {L}}S$? The following theorem says that, under very general conditions on the underlying density function, regardless of *d*, and as *n* goes to infinity, we can at least expect $25\%$ of the summands to be intervals.

### Theorem 5.1

Let $X_{1}, \dots , X_{n}$ be i.i.d. points taking values in ${\mathbb {R}}^{d}$, sampled from a common density function that is continuous almost everywhere with respect to the Lebesgue measure.

Consider a finite augmented metric space $(M = \{X_{1}, \dots , X_{n}\}, d_{M}, f)$, where $d_{M}$ is induced by the Euclidean metric in ${\mathbb {R}}^{d}$ and *f* is arbitrary, and let *S* be its density-Rips persistent set.

Let $\mathfrak {I}_{n}$ be the random variable that counts the number of intervals in the indecomposable decomposition of ${\mathcal {L}}S$, and let $\mathfrak {S}_{n}$ be the random variable that counts the total number of summands in the same decomposition. We have

3 $$\begin{aligned} \liminf _{n\rightarrow \infty } {{\,\textrm{E}\,}}\left[ \frac{\mathfrak {I}_{n}}{\mathfrak {S}_{n}}\right] \ge c(d), \end{aligned}$$

where *c*(*d*) is a constant that depends on *d*, and $c(1) = \frac{1}{3}$, $c(2) \approx 0.31$ and $c(d)\downarrow \frac{1}{4}$ as $d\rightarrow \infty$.

The rest of the section is dedicated to proving this theorem. The nearest neighbor graph of a metric space plays a fundamental role.

### Definition 5.2

The **nearest neighbor graph** of *M* is the directed graph on *M* given by the directed edges of the form $(x, x')$, where $x'$ is the nearest neighbor of *x*.

Now, we are interested in estimating the number of neighborly rooted elements, as in Definition 3.10, as they induce an interval in the decomposition of ${\mathcal {L}}S$. However, in general being neighborly rooted depends on *f*. To do without the condition on *f* we have:

### Lemma 5.3

Let $(M, d_{M}, f)$ be an augmented metric space and let *S* be its density-Rips persistent set. There are at least as many intervals in the indecomposable decomposition of ${\mathcal {L}}S$ as 2-cycles in the nearest neighbor graph of *M*.

### Proof

We can assume without loss of generality that $|{M}| \ge 2$. Let *G* be the nearest neighbor graph of *M*. The only cycles in this graph are precisely the 2-cycles, and each weakly connected component of *G* contains exactly one 2-cycle (see [25]).

Let $C_{1}, \dots , C_{k}$ be the weakly connected components of *G*. Fix $i \in \{1, \dots , k\}$, and let $x, y\in M$ be such that (*x*, *y*) and (*y*, *x*) is the 2-cycle in $C_{i}$. Either $f(y)\le f(x)$ or $f(x)\le f(y)$, and either *x* is neighborly rooted, *y* is neighborly rooted, or both are neighborly rooted. Say *x* is neighborly rooted, and define an endomorphism $\varphi _{i}:S\rightarrow S$ by setting

$$\begin{aligned} (\varphi _{i})_{p_{z}}(z) = {\left\{ \begin{array}{ll} S_{p_{y}\rightarrow p_{x}}(y), &  \text {if x = z,}\\ z, &  \text {otherwise,} \end{array}\right. } \end{aligned}$$

for every generator $z\in S_{p_{z}}$. Such an endomorphism is well-defined as shown in Proposition 3.5.

Constructing, for each *i*, an idempotent $\varphi _{i}$ as above, it is clear that we can iteratively peel off the associated intervals, yielding the desired conclusion. $\square$

Naturally, the number of 2-cycles is half the number of points that are the nearest neighbor of its nearest neighbor. The problem of estimating the probability for a point to be the nearest neighbor of its nearest neighbor, assuming a random point process, has been studied by multiple authors (see [21, 25–27, 34]).

In our case, when we have $X_{1}, \dots , X_{n}$ i.i.d. points in ${\mathbb {R}}^{d}$ sampled from a common density function under the conditions of Theorem 5.1, by [27, Thm. 1.1], and letting $N_{i,n}$ denote the probability event that $X_{i}$ is the nearest neighbor of its nearest neighbor, we have

4 $$\begin{aligned} \lim _{n\rightarrow \infty }{{\,\textrm{P}\,}}(N_{i,n}) = b(d), \end{aligned}$$

where *b*(*d*) is the volume of a unit *d*-sphere divided by the volume of the union of two unit spheres with centers at distance 1 (as in the magnified detail of Fig. 2b). In fact, $b(1) = \frac{2}{3}$, $b(2) \approx 0.621$, and $b(d)\downarrow \frac{1}{2}$ as $d\rightarrow \infty$ (see [34, Table 2]), and we define $c(d){:}{=} \frac{b(d)}{2}$.

We are now ready to finish the proof of Theorem 5.1 at the start of the section. Applying Lemma 5.3 and the linearity of expectation, it holds

$$\begin{aligned} {{\,\textrm{E}\,}}[\mathfrak {I}_{n}] \ge {{\,\textrm{E}\,}}\left[ \sum _{i=1}^{n}\frac{I(N_{i,n})}{2}\right] = \sum _{i=1}^{n}\frac{{{\,\textrm{E}\,}}[I(N_{i,n})]}{2} = \sum _{i=1}^{n}\frac{{{\,\textrm{P}\,}}(N_{i,n})}{2}, \end{aligned}$$

where $I(N_{i})$ is the indicator random variable of $N_{i,n}$. By (4) we have

$$\begin{aligned} \liminf _{n\rightarrow \infty } {{\,\textrm{E}\,}}\left[ \frac{\mathfrak {I}_{n}}{n}\right] \ge \frac{b(d)}{2} = c(d). \end{aligned}$$

Finally, we note that the number of summands in the decomposition is bounded by the number of points (the number of summands is bounded by the size of a minimal generating set). Thus, $\mathfrak {S}_{n}\le n$ and (3) of Theorem 5.1 follows, finishing the proof.
